# The Effect of Centralized Financial and Social Incentives on Cooperative Behavior and Its Underlying Neural Mechanisms

**DOI:** 10.3390/brainsci11030317

**Published:** 2021-03-02

**Authors:** Leticia Micheli, Mirre Stallen, Alan G. Sanfey

**Affiliations:** 1 Institute of Psychology, Leibniz University Hannover, 30159 Hannover, Germany; 2 Institute of Psychology, Leiden University, 2333 AK Leiden, The Netherlands; m.stallen@fsw.leidenuniv.nl or; 3 Amsterdam Research Institute for Societal Innovation, Amsterdam University of Applied Sciences, 1091 GH Amsterdam, The Netherlands; 4 Behavioral Science Institute, Radboud University Nijmegen, 6525 GD Nijmegen, The Netherlands; a.sanfey@bsi.ru.nl or; 5 Donders Institute for Brain, Cognition and Behavior, Radboud University Nijmegen, 6525 AJ Nijmegen, The Netherlands

**Keywords:** cooperation, public goods game, social and financial incentives, fMRI

## Abstract

Incentives are frequently used by governments and employers to encourage cooperation. Here, we investigated the effect of centralized incentives on cooperation, firstly in a behavioral study and then replicated in a subsequent neuroimaging (fMRI) study. In both studies, participants completed a novel version of the Public Goods Game, including experimental conditions in which the administration of centralized incentives was probabilistic and incentives were either of a financial or social nature. Behavioral results showed that the prospect of potentially receiving financial and social incentives significantly increased cooperation, with financial incentives yielding the strongest effect. Neuroimaging results showed that activation in the bilateral lateral orbitofrontal cortex and precuneus increased when participants were informed that incentives would be absent versus when they were present. Furthermore, activation in the medial orbitofrontal cortex increased when participants would potentially receive a social versus a financial incentive. These results speak to the efficacy of different types of centralized incentives in increasing cooperative behavior, and they show that incentives directly impact the neural mechanisms underlying cooperation.

## 1. Introduction

Cooperation is an integral characteristic of societies that function efficiently, with societal betterment generally best served by a willingness for individuals to make choices that benefit the public good, even though this may involve some personal sacrifice. For example, in the midst of the COVID-19 pandemic, individuals who wear masks effectively aim to cooperate in decreasing the spread of the disease and thus protect others as well. However, “free-riding”, that is, choosing to not cooperate oneself but still benefiting from the cooperation of others, is common. Individuals who refuse to wear masks in public spaces will still benefit from the cooperation and protection granted by others while not contributing themselves. Free-riding behavior is detrimental for society, as the benefits of slowing down contamination rates can only be achieved if everyone, or at least a critical mass of a population, decides to cooperate, and of course free-riding is often met with anger from those who are taken advantage of. To encourage cooperation, governments and institutions often employ incentives in order to guide behavior in societally beneficial directions. For instance, governments sometimes offer subsidies or tax breaks to citizens who use public transportation in order to stimulate their usage, but, conversely, availing of public transportation without an appropriate ticket can lead to a fine, thereby discouraging people to (literally) free ride on a public resource. Here, we explore how the looming threat of institutional (or centralized) incentives can guide cooperative behavior. Crucially, we extend previous research on this important question by experimentally examining scenarios that more closely mimic how these incentives are typically administrated in actual societal situations and by exploring the neural mechanisms underlying cooperation in the presence of such expected incentives.

The use and efficacy of rewards and punishments to both promote cooperation and reduce free-riding is supported by an extensive body of research. One stream of the literature has largely focused on the effect of rewards and punishments when these incentives are managed by peers (i.e., usually other participants in an experimental setting) [1,2,3,4,5]. Experiments have shown that individuals typically cooperate when they expect others to be cooperative as well [6]. Violations of these expectations often lead cooperative individuals to punish free-riders, even when doing so is costly [3,4] and when chances of interacting with the same free-riders in the future are negligible [7]. Likewise, individuals also reward others for being cooperative [1,2]. Most importantly, peer incentives are effective in promoting cooperation [4,8], given that individuals tend to become more cooperative (or keep being cooperative) after being incentivized by their peers (for a review, see [9]).

Despite the important contribution of this literature to the understanding of the evolution of cooperation, peer incentives can only function under certain conditions, for example when the group size is rather small [10]. As societies grow in size, the application of incentives is transferred to a separate authority [11], and thus societal incentives are typically administered by a non-peer, centralized source such as a government or an employer [10]. Although there are differences in how individuals react to incentives when they are peer-administered as opposed to centralized [2,12], both types of incentives have been shown to promote cooperative behavior [1,2,3,4,5,8,10,12,13,14,15]. Given that incentives are often, and increasingly, administered by governments and institutions in our current societies, here, we focus our attention on the use of centralized incentives (as opposed to incentives administered by peers). In doing so, we explore the role of different types of centralized incentives in the promotion of cooperation, as well as investigating their underlying neural mechanisms.

Importantly, although studies employing a peer system of incentive delivery have shown that both financial and non-monetary sanctions and rewards can increase cooperation rates [16,17,18,19], most experiments studying the effect of centralized incentives on cooperation have focused solely on financial rewards and punishments in an effort to shape behavior. However, society also implements, and indeed increasingly uses, social incentives, which typically operate via more affective mechanisms rather than the reward trade-offs encouraged by financial incentives. For instance, streets and buildings can be named after people who performed a public good, and some companies make use of social rewards such as “employee of the month” to acknowledge workers who have stood out in their contributions to the company. Moreover, free-riders are sometimes named and shamed in the media (as an example, the UK government publishes annually a list containing the names of tax evaders) and some initiatives publicize rankings of companies based on their contributions (or lack thereof) to society (e.g., the Corporate Social Responsibility Monitor). The dramatic rise in social media use also allows for reputations to be either enhanced or damaged very easily and rapidly. Research has shown that reputational concerns can indeed be powerful tools in promoting norm compliance and cooperation [16,20,21,22]. Mere cues of being observed can already induce prosociality [23,24], and incidental emotions such as shame have been shown to foster cooperation among self-interested individuals [25]. In fact, while internal predispositions toward social comparison and social approval can already promote prosocial behavior [26], the employment of social rewards and punishments by external institutions can increase prosocial behavior even more. Thus, we expect that even though both centralized financial and social incentives may promote cooperation, they may differ in their motivational underpinnings, with social incentives relying more strongly on affect and internal predispositions to social comparison and approval.

An additional important difference in how the effect of incentives on cooperation is typically studied experimentally versus how they are employed in real-life scenarios is that in most laboratory studies, incentives are applied immediately (and deterministically) in order to examine how behavior changes *after* the implementation of incentives. This implies that the maintenance of cooperation relies on rewards and punishments being administered right after cooperation and defection, respectively [12]. However, in everyday life, the delivery of incentives is usually probabilistic. For instance, riding the bus without a valid ticket or littering in public does not necessarily always result in a fine. Moreover, many individuals may display cooperative (or non-cooperative) behavior even though they may have never been personally rewarded or punished. Therefore, in order to investigate how incentives work in more naturalistic and ecologically valid scenarios, where presumably affect plays a more central role, incentives in the present study are not applied directly during the experiment, but only afterwards. This allows us to explore whether the prospect of reward or the looming threat of punishment may already be effective in changing behavior. Specifically, in our study, incentives are applied in a probabilistic fashion. That is, individuals know that their behavior may not always be rewarded or punished at the end of the experiment, as incentives are only administered with certain probabilities, which is an approach that has also been shown to be efficient in terms of costs to societies [27].

A final goal of the present research was to explore the associated neural processes underlying cooperation when centralized financial and social incentives were present. We used functional magnetic resonance imaging (fMRI) to scan participants as they made cooperative or non-cooperative decisions under the possibility of either being rewarded or punished by a centralized source depending on their level of contribution to the group. Although different processes have been associated with cooperative behavior, such as reward-processing [28,29,30,31], compliance with internal social norms [32,33], and mentalizing [34,35], less is known about how these processes are influenced by the introduction of (probabilistic) incentives [36]. The use of modern neuroimaging techniques offers a useful tool to gain additional understanding of the motives behind cooperation [37], thus better informing what psychological processes are impacted by potential future incentives and also potentially yielding more insight as to how these incentives can be usefully employed in society.

Previous studies have shown that in the presence of threats of peer punishment, brain regions associated with cognitive control processes are important in changing behavior toward cooperation [32,38,39]. However, a recent fMRI meta-analysis attempting to characterize the brain mechanisms of prosocial choice in the presence of incentives has pointed out inconsistencies in how the regions associated with social cognition are linked to behavior when incentives are present or not, warranting further studies [40]. Importantly, we note that previous efforts to examine the neural mechanisms of incentives and behavior have concentrated almost exclusively on the effect of peer-delivered incentives. The present research aims to provide more insight into the effect of centralized incentives on cooperation and its neural mechanisms, thereby exploring whether financial incentives and social incentives impact cooperation and its underlying mechanisms in a similar way or whether their effects differ.

To address the aforementioned limitations in earlier work, we developed an experimental paradigm to investigate the effect of centralized incentives on cooperation when (a) incentives took either financial or social forms; (b) individuals did not directly receive incentives but were only affected by the prospect of punishment or reward; and (c) individuals were undergoing fMRI. This novel task is based on the well-known Public Goods Game (PGG) [3] and employed a within-subject design to allow for a direct comparison between the impact of different types of social and financial centralized incentives. Specifically, participants were exposed to different trial types in which cooperation could be incentivized via financial rewards or punishments, social rewards or punishments, or where potential incentives of any kind were absent. We used the same paradigm for both a behavioral study (Study 1) and a neuroimaging study (Study 2) to also allow for testing the replicability of experimental effects across two studies with different samples and different settings (behavioral laboratory vs. MRI environment).

## 2. Study 1: How the Prospect of Centralized Financial and Social Incentives Impact Cooperation

### 2.1. Materials and Methods

#### 2.1.1. Participants

Fifty-six participants (*M_age_* = 21.2 years, *SD* = 2.66 years, 43 female) were recruited from Radboud University Nijmegen, The Netherlands. Participants received a standard participation fee of either one credit (*N* = 23) or €8 (*N* = 33), with an additional bonus of a maximum of €8 dependent on their performance in the task. On average, participants earned €10.20 (*SD* = €4.10) in total.

#### 2.1.2. Experimental Procedure

Participants provided written informed consent upon arrival according to procedures approved by the local ethical committee. Additionally, they were asked for permission to have their identity and subsequent task performance reported to the full participant group of the study. Participants who agreed to have their identity revealed emailed a photograph of themselves to the experimenter upon study completion. Four participants who did not consent to reveal their identity were subsequently excluded for analyses, as their anonymity in the task could presumably influence their decisions. Therefore, the final sample of participants consisted of fifty-two participants (*M_age_* = 21.19 years, *SD* = 2.63 years, 39 female).

Participants received written instructions about the experimental task and answered several quiz questions in order to ensure all instructions were understood correctly. Prior to the start of the experimental task, participants’ answers in the quiz questions were checked by the experimenter, and participants were provided the opportunity to ask any remaining questions with regard to clarifying the task. Participants performed the experimental task, developed with E-Prime software 2.0, in a computer cubicle. After the experimental session, participants completed questionnaires assessing individual differences in risk aversion, social comparison tendencies, and their expectations about the choices of the other participants in the PGG (see section “Questionnaires” below). The experiment lasted for approximately 1 h.

#### 2.1.3. Public Goods Game with Centralized Incentives

In order to examine the effect of centralized incentives on cooperative behavior, participants played a modified version of the Public Goods Game. Participants were randomly assigned to a group consisting of four members and received an endowment in tokens. Subsequently, they were asked how much (if any) of these tokens they wanted to contribute to a group pot. Tokens that were placed in the group pot were multiplied by a factor of 1.6. Then, the resulting amount was equally distributed between participants (each therefore received a quarter of the group pot). Participants were free to contribute as much as they wanted, and participants kept the tokens that were not invested into the group pot for themselves. At the end of each trial, participants received the number of tokens they had kept for themselves, plus their share of the tokens from the group pot. Participants were informed that all group members received the same endowment and faced the same decision to contribute or not to the group pot. Moreover, participants were told that during the task, all group members (including themselves) would remain anonymous and that they would never interact with the same group more than once. Importantly, and in contrast to a typical Public Goods Game, our task consisted of five trial types—trials in which financial incentives were used to either potentially reward cooperation or punish non-cooperation, trials in which social incentives were used to either potentially reward cooperation or punish non-cooperation, and trials in which incentives were absent. Trial types were presented randomly, and participants played all five of them.

##### Financial and Social Incentives

In both the financial reward and punishment trial types, participants could receive a monetary bonus or fine, depending on their contribution to the group pot. Specifically, in the financial reward trials, participants could receive additional bonus tokens if their contribution to the group pot was higher than the average contribution of the group, while in financial punishment trials, participants could lose tokens if their contribution to the group pot was lower than the average group contribution for that trial. Tokens were converted to Euros at the conclusion of the experiment.

**Figure 1:**
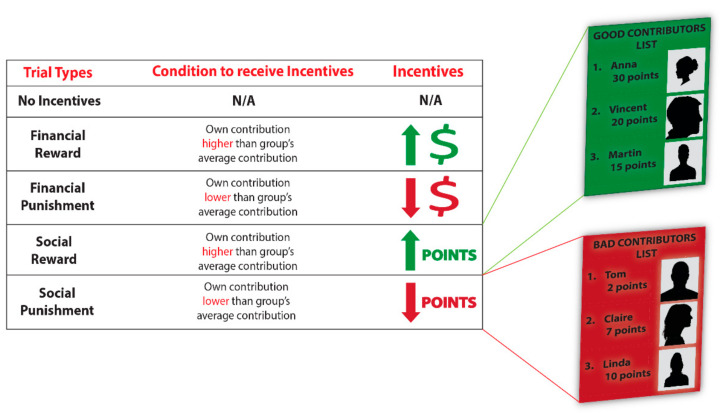
Overview of trial types. In the financial and social conditions, participants could receive incentives (rewards or punishments) based on their contributions to the group account. In the financial condition, these incentives were monetary, while in the social condition, these incentives were social, consisting of rankings on the Good or Bad Contributors List (based on participants’ performance in social reward and punishment trial types respectively). Participants’ rankings on the Good and Bad Contributors lists were calculated after all data were collected and were only shown to participants in this specific experiment after study completion.

In the social reward and punishment trial types, incentives were administered according to the same set of rules; however, the nature of the incentive was now social instead of monetary. In social reward trials, participants could receive “List Points” for cooperation, while in social punishment trials, participants could lose “List Points” for not cooperating. These List Points determined participants’ ranking on two lists that would be shown to all participants at the conclusion of the experiment—a “Good Contributors List” and a “Bad Contributors List”. These lists displayed participants’ names, photographs, and their final point scores in the PGG. Those participants who ended up with the highest number of “List points” were displayed on the “Good Contributors List”, while those who scored the lowest number of points were shown on the “Bad Contributors List”. That is, the more points a participant earned in social reward trials, the higher his/her position on the “Good Contributors List”, whereas the more points a participant lost in social punishment trials, the higher his/her position on the “Bad Contributors List”. Importantly, each participant belonged to one list only. Both lists were made visible to participants after all data collection was complete and were published on a secure website for a period of two weeks. Participants in the experiment were provided with a private username and password to access the website. See Figure 1 for an overview of the trial types in the experiment.

##### Probabilistic Nature of Incentives

Although participants knew that they could gain or lose tokens (in the financial condition) and points (in the social condition) depending on their choices, feedback on participants’ decisions was not provided during the experiment. This was because we were interested in studying whether the mere threat of punishment or hope of reward could already influence decisions. To this end, participants were not informed about the average group contribution amount in each trial. Therefore, they could not know in each trial whether they would earn a reward or a punishment. Moreover, they did not know if the incentive would actually be administered on a given trial nor how large the incentive would be. To reflect the uncertain nature of incentives in real life, participants were told that a pre-determined percentage of the trials would be incentivized. Additionally, to prevent participants from being distracted by calculating potential outcomes and adopting certain strategies as a result, participants were told that the size of the incentive was also variable and would range between 50 and 80% of participant’s total income on the selected trial. In line with how incentives for cooperation are typically administered by an external source (e.g., the government or an employer), incentives in the PGG were always administered by the experimenter and not by other participants in the game.

Importantly, individual’ choices impacted the actual payout of other participants in the experiment. At the conclusion of the study, we determined participants’ payment by randomly pairing their choices with the choices of other experimental participants. This incentive structure allowed us to make the PGG realistic and engaging, and it ensured that participants’ decisions in each and every trial were equally likely to be consequential for their own payout as well as for those of others. At the end of the study, 15 trials (3 from each trial type) were randomly selected to determine participants’ payoff. For each of these trials, we added the number of tokens participants did not invest in the group pot to the number of tokens returned from the group pot on the selected trial. For the financial reward and punishment trials, additional tokens were then added or subtracted if participants had been selected to receive a reward or punishment, respectively. However, even when financial incentives were absent, participants were still incentivized to respond meaningfully, given that all decisions could impact their payment. The final number of tokens were converted to Euros (1 token/1.5 Euro cent) and added to participants’ baseline payment amount.

##### Trial Outline

**Figure 2:**
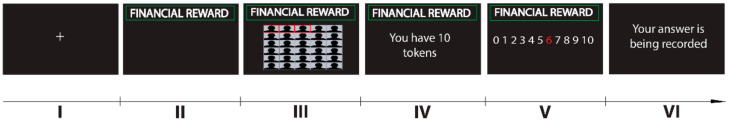
Trial outline. The beginning of each trial was indicated by a fixation cross, followed by a screen revealing the upcoming trial type (screen II). Participants were paired with three randomly selected other people from the participant pool (screen III). Screen IV showed how many tokens participants received, and on screen V, participants were asked to indicate how many tokens they liked to contribute to the group pot. The final screen VI showed that the participant’s choice was being processed.

The PGG consisted of 75 trials: 15 in the no incentive condition, 30 in the financial condition (15 financial reward, 15 financial punishment), and 30 in the social condition (15 social reward, 15 social punishment). Each trial began with a fixation cross, after which participants saw a screen announcing the upcoming trial type. Trial type was indicated by a colored frame displayed on top of the screen. Next, group members were selected, and participants were shown their endowment for that trial; then, they were asked how much they wanted to contribute to the group account (response window of 10 *s*). Participants were endowed with either 10, 20, or 30 tokens on each trial (selected randomly) in order to encourage them to think carefully about each decision and minimize any possible automatic responses. Participants indicated their responses by using 2 buttons to scroll either left or right across a response bar with numbers ranging from 0 to their endowment on that trial (10/20/30), with a third button used to confirm their choice. On each trial, the starting point in the response bar from which participants could scroll left or right was randomly chosen. To ensure that participants always had the same number of options to choose from, response options were displayed in steps of either 1 token (on trials with a 10 token endowment), 2 tokens (20 token endowment), or 3 tokens (30 token endowment). Each trial concluded with a screen indicating that the response had been recorded. See Figure 2 for an overview of the trial outline.

#### 2.1.4. Questionnaires

##### Expectations

After the task, participants were asked how much they believed other players contributed in the PGG. Participants were asked to indicate their answer in percentages of total endowment for each of the three conditions (social, financial, and no incentives).

##### Lottery Choice Task

Investing in the group pot can be perceived as risky, since participants were uncertain about the amount of money that would be returned, as this amount depended on the investments of others as well. In order to gain more insight into the effect of potential individual differences in financial risk preferences on contribution amounts, participants completed the Ten Paired Lottery-Choice Decisions with low payoffs [41].

##### Social Comparison

Social incentives may be more salient to individuals who are more inclined to compare themselves with others. To test whether individual differences in social comparison tendencies correlated with participants’ willingness to contribute in the social conditions of the PGG and their response to incentives, participants completed the Social Comparison Orientation scale [42].

#### 2.1.5. Behavioral Analyses and Statistics

Data analyses were performed using the R project for statistical computing [43]. First, we compared behavioral responses when probabilistic incentives were present versus absent. The dependent variable of interest was the percentage of tokens that participants contributed to the group pot in each trial. Presence of probabilistic incentives (2 levels: no incentives and incentives, with financial and social incentives collapsed) and endowment (3 levels: 10, 20 and 30 tokens) were included as within-subject variables as well as an interaction term for the presence of incentives and endowment level. The baseline was defined as the condition in which no probabilistic incentives were present and the endowment equaled 10 tokens. Two-tailed multilevel linear model analysis was performed using the lme4 package in R [44]. To account for the repeated-measures structure of the data, the model allowed for a random intercept to vary per participant. Post-hoc analyses were conducted using the ‘difflsmeans’ function, and P values were obtained using Kenward–Roger corrections through the lmerTest package in R [45]. Second, to get more insight into how different incentive types affected cooperative behavior, a second multilevel linear model was fitted including condition (3 levels: financial, social, and no incentives) as a within-subject factor with a random intercept and varying slope per participant and condition, respectively. The baseline was again defined as the no incentive condition. The dependent variable was the same as in the previous model. Lastly, to gain more understanding into the effect of probabilistic punishments and rewards on cooperation, we fit a model including Trial types (4 levels: financial reward, financial punishment, social reward, and social punishment) as a within-subject factor again with a random intercept and slope varying per participant and trial type, respectively. The dependent variable was the same as in the previous models. Correlational analyses were performed between participants’ contributions in the different conditions of the PGG and their individual scores on the questionnaires measuring their expectations about the choices of other participants, their risk aversion scores, and their social comparison scores.

### 2.2. Results

Results of our first multilevel model analysis including the presence of probabilistic incentives (no incentives vs. financial/social incentives collapsed together) and endowments as within-subject variables revealed a significant main effect of the presence of probabilistic incentives on the percentage of tokens contributed to the group pot, with participants contributing significantly more when incentives were present compared to absent (F(1, 3827) = 752.7, *p* < 0.001; Incentives present: *M* = 0.62, *SE* = 0.02, Incentives absent: *M* = 0.36, *SE* = 0.02). Although not hypothesized a priori, we found a significant main effect of endowment (*F*(2, 3827) = 11.52, *p* < 0.001), with participants contributing most when the endowment was the lowest (*M_Endowment of 10 tokens_* = 0.51, *SE* = 0.021, *M_Endowment of 20 tokens_* = 0.49, *SE* = 0.021, *M_Endowment of 30 tokens_* = 0.47, *SE* = 0.021, Least squared differences: 10 vs. 20 tokens: *t*(3825) = 1.94, *p* = 0.09; 10 vs. 30 tokens: *t*(3825) = 3.95, *p* < 0.001; 20 vs. 30 tokens: *t*(3825) = 2.0, *p* = 0.09, corrected for multiple comparisons with the Holm method). There were no interaction effects between the presence of incentives and endowment level (*p* = 0.98). Additionally, results did not change when including gender or the type of payment participants received in exchange for their participation (i.e., credits vs. money) as between-subjects variables; that is, there were no differences in cooperation rates between males and females (*M_Male_* = 0.5, *SE* = 0.04, *M_Female_* = 0.49, *SE* = 0.024, Least squared differences: Male vs. Female: *t*(49) = 0.22, *p* = 0.83), or between participants who received money or credits in exchange for their participation (*M_Points_* = 0.49, *SE* = 0.03, *M_Money_* = 0.49, *SE* = 0.029, Least squared differences: Points vs. Money: *t*(49) = 0.03, *p* = 0.98).

**Figure 3:**
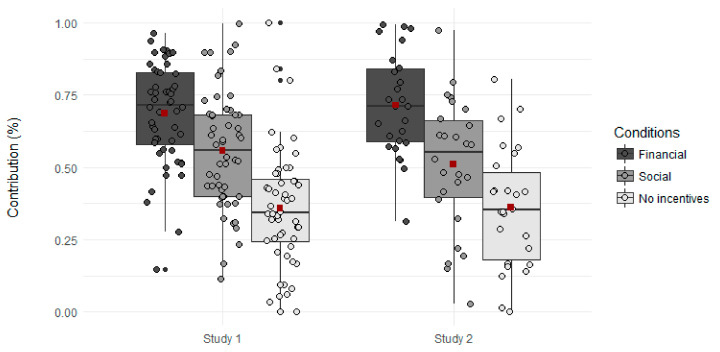
Effect of incentive condition on contributions to the group pot in Study 1 and Study 2. Contributions rates were significantly higher in the incentivized conditions than in the no incentive condition, with contributions being highest in the financial condition. Medians are represented by the middle line within the box-plots, whereas the red squares display means per condition. Individual data points are shown per condition.

To examine the effects of incentive type on cooperation, a second multilevel linear model was run with condition (financial, social, no incentives) as the within-subjects variable and the percentage of tokens contributed to the group pot as the dependent variable. We found that participants contributed most in the financial condition and least when no incentives were present (see Figure 3, left panel) (*F*(2, 51) = 46.25, *p* < 0.001; Financial: *M* = 0.69, *SE* = 0.024, Social: *M* = 0.56, *SE* = 0.028, No incentives: *M* = 0.36, *SE* = 0.029; Least squared differences: Financial vs. No incentives: *t*(51) = 9.39, *p* < 0.001, Social vs. No incentives: *t*(50.8) = 6.92, *p* < 0.001, Financial vs. Social: *t*(51) = 4.23, *p* < 0.001, corrected for multiple comparisons with the Holm method).

A third and final multilevel linear model was conducted to explore the effect of rewards and punishments on cooperation, with Trial Type (financial reward, financial punishment, social reward, and social punishment) as a within-subject factor and the percentage of tokens contributed to the group pot as the dependent variable. There were no significant differences between the effect of reward and punishment on contribution amount (Least squared differences: Financial punishment vs. Financial reward *t*(51) = −1.73, *p* = 0.09; Social punishment vs. Social reward: *t*(51) = −0.83, *p* = 0.41). The inclusion of endowment in the second and third models described above did not change the results.

A Pearson correlational analysis showed that participants’ self-reported expectations about the behavior of others significantly correlated with their contribution in the no incentive, financial, and social conditions (No incentives: *r* = 0.74, *p* < 0.001; Financial: *r* = 0.77, *p* < 0.001; Social: *r* = 0.62, *p* < 0.001). That is, the more participants expected others to cooperate and invest in the group pot, the more they contributed themselves. Individual measures of risk aversion and social comparison did not correlate with contribution amounts (Risk aversion: No incentives: *r* = 0.12, *p* = 0.82; Financial: *r* = −0.21, *p* = 0.39; Social: *r* = −0.03, *p* = 0.82, all comparisons corrected for multiple comparisons with the Holm method; Social Comparison: Social: *r* = 0.08, *p* = 0.57).

### 2.3. Conclusions

In Study 1, we investigated how the prospect of receiving centralized probabilistic incentives, either of a financial or social nature, impacted cooperative behavior in comparison to situations in which those incentives were not available. The results of Study 1 demonstrated that both the possibility of receiving financial and social incentives increased cooperation levels as compared to when no incentives were present, with financial incentives having a larger effect on contribution amounts than social incentives. Importantly, our implementation of incentives here was probabilistic, in that participants were never actually sure whether incentives would be enacted on a specific trial or even knew the precise size of the incentives. Moreover, participants did not receive any rewards or punishments during the task, illustrating that merely the potential hope or threat of incentives can play a significant role in enhancing cooperation. In addition, and importantly, the social incentive condition, that is, one that is (more or less) “free” to the incentive provider given low or absent implementation costs, yielded significantly increased rates of cooperation as compared to when incentives were not provided. Study 2 was designed to replicate the behavioral effects found in Study 1 and to shed light on the neural mechanisms underlying these effects of centralized incentives on cooperation.

## 3. Study 2: The Neural Bases of the Prospect of Centralized Incentives on Cooperation

### 3.1. Materials and Methods

#### 3.1.1. Participants

Thirty right-handed participants (*M_age_* = 23.6 years, *SD* = 4.02 years, 18 female) who did not take part in Study 1 were recruited from Radboud University Nijmegen. Participants received a standard participation fee of either 2.5 credits (*N* = 3) or €25 (*N* = 27), with an additional bonus of maximum €8 based on their task performance. On average, participants earned €28 (*SD* = €7.46) in total. For the same reasons as in Study 1, we excluded four participants who did not consent to reveal their identities in the Good and Bad Contributors Lists. Therefore, the final sample for Study 2 consisted of 26 participants (*M_age_* = 23.7 years, *SD* = 4.2 years, 15 female).

#### 3.1.2. Experimental Procedure

The same experimental procedure as in Study 1 was followed. Participants were asked for permission to have their identity and subsequent task performance reported to the full participant group of the study. In Study 2, participants performed the PGG while undergoing MRI. Nevertheless, to ensure all participants fully comprehended the experimental task, participants were asked to read the instructions and answer quiz questions about the experiment before entering the scanner. As in Study 1, participants’ answers were checked by the experimenter and participants were given the opportunity to ask any remaining clarification questions before the start of the experimental task. The PGG was divided into 2 blocks of approximately 30 min each to provide subjects with a small break halfway through the task. After the scanning session, participants completed the same questionnaires as in Study 1. The total duration of the experiment was ≈150 min.

#### 3.1.3. Public Goods Game with Centralized Incentives

The PGG was slightly adapted for use in the MRI scanner. The number of trials was increased in Study 2, resulting in 105 trials (21 per trial type). Each trial started with a fixation cross (duration varied between 2 and 5 s), after which participants saw the upcoming trial type (2 s). Then, group members were selected (4 s), participants were shown their endowment for that trial (4 s), and then, they were asked how much they wanted to contribute to the group account (response window of 6 s). Participants indicated their responses in a button box compatible with fMRI. They used 2 buttons to scroll either left or right over the response bar and a third button to confirm their choice. Each trial concluded with a screen indicating that the response had been recorded (3 s).

Importantly, as data acquisition took several months, we determined participants’ bonus at the end of each experimental session by pairing their choices with the decisions of three randomly selected participants of Study 1. We repeated this procedure for each of the fifteen trials that were randomly selected for payout. Participants received their bonus via bank transfer within two weeks of study participation.

#### 3.1.4. MRI Acquisition Parameters

A 3T Siemens Skyra scanner was used for imaging data acquisition. Functional images were acquired in two runs using an ascending slice acquisition and a T2*-weighted multiple echo planar imaging (MEPI) sequence (TR = 2.25 s, TE = 9.4 ms, flip angle = 90°, slice matrix = 64 × 64 mm, slice thickness = 3 mm, slice gap = 0.5 mm, FOV = 224 mm). Structural images were acquired after the functional runs with a T1-weighted MPRAGE sequence (192 sagittal slices, TR = 2.3 s, TE = 3.03 ms, flip angle = 8°, slice matrix = 256 × 256 mm, slice thickness = 1 mm, slice gap = 0.5 mm, FOV = 256 mm).

#### 3.1.5. Behavioral Analyses

The behavioral data were analyzed as described in Study 1 above.

#### 3.1.6. Imaging Analyses

Imaging data were preprocessed and analyzed with the SPM8 software package [46]. To allow for longitudinal relaxation time equilibration, the first 29 volumes of the first echo sequence as well as the first 3 volumes of the echo sequence of the second run were discarded. Functional images were corrected for slice scan time, realigned to the first scan pulse of the corresponding run, and then coregistered to each participant’s high-resolution structural scan. Structural scans were segmented into gray and white matter. Functional images were normalized to the Montreal Neurological Institute (MNI) template. Images were smoothed with a Gaussian kernel of 8mm fill-width at half-maximum to reduce noise.

We estimated two first-level general linear models, with the first model focusing on the neural activity associated with the presentation of trial type (GLM 1: brain responses time-locked to the presentation of screen II; see Figure 2), and the second model focusing on the neural activity associated with participants’ choices (GLM 2: brain responses time-locked to the presentation of screen V; see Figure 2). In model 1, the event of interest was modeled with a duration of 2 s, which was equal to the screen presentation time. In model 2, the modeled duration equaled participants’ reaction time (i.e., time between the moment participants first saw screen V and the moment they confirmed their choice; *M* = 2.64 s, *SD* = 1.05 s). In both models, incentive conditions (none, financial, social) were defined as regressors of interest, and motion parameters were included as nuisance variables. Regressors were modeled with a canonical hemodynamic response function. For model 2, participants’ percentual contribution amounts on a trial basis were standardized per condition and transformed into z-scores, and they were included as parametric modulators for the regressors of interest (orthogonalized to the unweighted regressors). All reported activations survived a threshold corresponding to *p* < 0.05 family-wise error (FWE) corrected for multiple comparisons at the cluster level, with an underlying threshold corresponding to *p* < 0.001 at the whole-brain level (uncorrected; *k* > 10).

### 3.2. Results

#### 3.2.1. Behavioral Results

The behavioral findings of Study 1 were replicated in Study 2. Results of an initial multilevel linear model analysis, with the same specifications as in Study 1 and including the presence of incentives (no incentives vs. financial/social incentives collapsed), endowments, and the interaction between the presence of incentives and endowment level as within-subject variables, showed a main effect of incentives on the percentage of tokens contributed to the group pot, with significantly higher contributions when incentives were present as compared to when they were absent (*F*(1, 2660) = 531.1, *p* < 0.001; Incentives present: *M =* 0.61, *SE* = 0.03, Incentives absent: *M* = 0.36, *SE* = 0.03). The main effect of endowments found in Study 1 was also replicated in Study 2 (*F*(2, 2660) = 8.4, *p* < 0.001) with higher contributions the lower the endowment was. However, in contrast to Study 1, the comparison between 20 and 30 tokens was no longer marginally significant (*M_Endowment of 10 tokens_* = 0.51, *SE* = 0.032, *M_Endowment of 20 tokens_* = 0.48, *SE* = 0.032, *M_Endowment of 30 tokens_* = 0.47, *SE* = 0.032, Least squared differences: 10 vs. 20 tokens: *t*(2658) = 2.12, *p* = 0.06; 10 vs. 30 tokens: *t*(2658) = 3.01, *p* = 0.008; 20 vs. 30 tokens: *t*(2658) = 0.89, *p =* 0.37, corrected for multiple comparisons with the Holm method). Furthermore, there was no interaction between presence of incentive and endowment level (*p* = 0.70). As in Study 1, we found that controlling for the type of payment participants received in exchange for their participation did not impact the results, and contribution rates did not differ according to compensation type (*M_Points_* = 0.49, *SE* = 0.03, *M_Money_* = 0.49, *SE* = 0.09, Least squared differences: Points vs. Money: *t*(23) = 0.02, *p* = 0.98). Controlling for gender also did not change results, and contribution rates did not differ between male and female participants (*M_Male_* = 0.54, *SE* = 0.06, *M_Female_* = 0.44, *SE* = 0.05, Least squared differences: Male vs. Female: *t*(23) = 1.61, *p* = 0.12). To examine the effect of the type of incentive on cooperation, a second multilevel linear model was run with conditions (financial, social, no incentives) as a within-subjects variable and the percentage of tokens contributed to the group pot as the dependent variable. We found that participants contributed the most when financial incentives were possible and least when probabilistic incentives were absent (*F*(2, 25) = 44.06, *p* < 0.001; Financial: *M* = 0.71, *SE* = 0.035, Social: *M* = 0.51, *SE* = 0.045, No incentives: *M* = 0.36, *SE* = 0.041; Least squared differences: Financial vs. No incentives: *t*(25) = 9.00, *p* < 0.001, Social vs. No incentives: *t*(25) = 2.8, *p* = 0.009, Financial vs. Social: *t*(25) = 4.5, *p* < 0.001, corrected for multiple comparisons with the Holm method); see Figure 3, right panel.

Finally, in a third multilevel linear model with trial type (financial reward, financial punishment, social reward, and social punishment) as a within-subject factor and percentage of tokens contributed to the group as the dependent variable, we found that contribution amounts were not impacted by whether the incentives were administered as rewards or punishments (Financial punishment vs. Financial reward: *p* = 0.35; Social punishment vs. Social reward: *p* = 0.21). The inclusion of endowment in the second and third models reported above did not change the results.

We also found that across conditions, contributions were positively correlated with participants’ expectations about the behavior of others, with participants contributing more when they expected others to contribute more (No incentives: *r* = 0.75, *p* < 0.001; Financial: *r* = 0.74, *p* < 0.001; Social: *r* = 0.42, *p* = 0.03). Individual measures of risk aversion and social comparison did not correlate with contribution amounts (Risk aversion: No incentives: *r* = −0.27, *p* = 0.56; Financial: *r* = −0.02, *p* = 1; Social: *r* = 0.1, *p* = 1; all comparisons corrected for multiple comparisons with the Holm method; Social Comparison: Social: *r* = −0.1, *p* = 0.59).

#### 3.2.2. Functional Imaging Results

**Figure 4:**
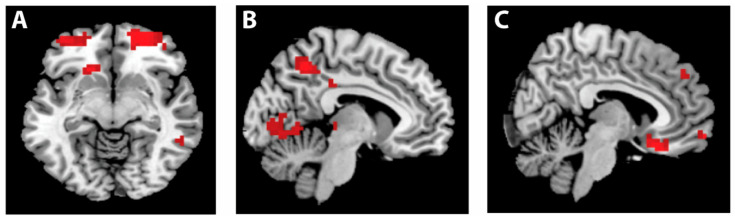
Neuroimaging results. (**A**). Bilateral lateral orbitofrontal cortex (*z* = −8) was activated more in trials in which incentives were absent vs. present (screen II). (**B**). Activation in the precuneus (*x* = −8) was associated with the absence of incentives during the moment of choice (screen V). (**C**). The right medial orbitofrontal cortex (*x* = 6) showed more activation when social incentives could be received compared to financial incentives during the moment of choice (screen V).

To examine how the provision of centralized incentives can impact cooperation rates, we first analyzed the neural data associated with the prospect of either simply potentially receiving incentives or not (screen II), that is, comparing trials in which incentives were absent with trials in which incentives were possible, and vice versa (collapsed across social and financial incentives; GLM 1). This analysis yielded activity in the bilateral lateral orbitofrontal cortex (lOFC), which was significantly greater when incentives were absent as compared to present (see Figure 4A, Table 1). There was no significant activity for the reverse contrast (incentives present vs incentives absent).

To further explore the neural activity underpinning contribution decisions in the absence and presence of incentives, we compared the BOLD signal at the time of decision in trials in which incentives were absent versus present (collapsed over social and financial incentives, screen V, GLM 2) controlling for contribution amount across conditions. This analysis showed greater activation in the precuneus when incentives were absent compared to present (Figure 4B, Table 2). The reverse contrast (incentives present vs. incentives absent) did not yield significant activity.

Next, to address our question regarding how incentive type itself might play a role in cooperative decisions, we compared neural activity between trials with probabilistic social incentives and trials with financial incentives, again controlling for contribution amounts across conditions. Analyses demonstrated significantly increased activation at the time of decision in the right medial orbitofrontal cortex (mOFC) when cooperation was incentivized by social as compared to financial incentives (Figure 4C, Table 2, GLM 2). The reverse contrast yielded no significant results. No differences were found between social and financial incentives in the anticipation phase of the decision-making (GLM 1).

#### 3.2.3. Conclusions

In Study 2, we replicated the behavioral findings of Study 1, supporting the conclusion that the mere promise/threat of incentives is already effective in promoting cooperation. Neuroimaging results provided insights into how these centralized incentives may act, showing that activation in the lOFC and precuneus was higher in the absence of incentives, that is, when the decision to cooperate was likely based on an internal prosocial motivation, as opposed to the possibility of an additional incentivized gain or loss in either the social or financial domain. Regarding the comparison of different incentive types, imaging data here showed that during the decision phase of the PGG, there was increased activation of right mOFC when considering social incentives as compared to financial incentives.

## 4. General Discussion

Given that in daily life, many of our societal incentives are typically administered by a centralized authority, whether it be a government or an employer, here, we investigated how centralized financial and social incentives can impact cooperative decision-making, both in terms of how the provision of different types of incentives can motivate cooperation as well as exploring the associated underlying neural processes. In line with previous studies on centralized incentives [10,13,14,15,47], results across two separate samples of participants and across two different testing contexts (i.e., behavioral laboratory and fMRI scanner) demonstrated that centralized rewards and punishments can indeed promote cooperative behavior.

An important point to note is that in the present research, participants did not receive punishments or rewards during the task, as we were interested in the effect of the prospect of incentives in modulating cooperative behavior. To this end, we used probabilistic incentives, that is, participants never knew whether a given trial would be a candidate for an extra reward or punishment, or even what the likelihood of reward or punishment was overall. Nonetheless, our results indicate that the mere threat of punishment or hope of reward was sufficient to increase contribution rates significantly, indeed leading to them almost doubling. This has some clear implications for the efficient use of incentives in real-life decision-making, as probabilistic incentives decrease the costs to institutions associated with punishing and rewarding individuals while not affecting their trust in the incentive system in place [27]. In fact, our results suggest that even the possibility that a cooperative act could be rewarded (or a non-cooperative act could be sanctioned) is apparently adequate to see an increase in public good contributions.

Additionally, and in line with previous studies investigating the effect of peer-delivered social incentives on cooperation [16,17], our results show that the provision of social incentives has a strong effect on cooperation when incentives are centralized, yielding significant increases in cooperation as compared to the no incentive condition. Here, participants voluntarily chose to make their identity and task performance visible to others, and the subsequent presence of a ranked list of contributors was sufficient to induce rather substantial increases in cooperative choices. These results resonate with previous work suggesting the role of affect and moral emotions such as shame and honor in driving cooperative behavior [25,48]. During conditions in which social incentives were present in our studies, participants might have anticipated the emotions they would feel when their behavior would become eventually observable by others and adjusted their behavior toward cooperation. As well as demonstrating the clear role of affect in driving social interactive decision-making, we believe this is an important finding, especially given the rise and ubiquity of social media, as it provides a potentially powerful, low-cost, avenue for increasing reputational concerns and prosocial behavior. Of course, these approaches should be used with caution, as they also have the potential to impact less positive behavior, but nonetheless, our results clearly demonstrate the potential power of social comparison in facilitating socially desirable behavior. However, it is worth noting that although social incentives increased cooperation compared to instances when incentives were absent, the increase in cooperation was higher in the presence of centralized financial incentives than in the presence of social incentives. This finding corroborates previous work comparing the effect of peer-delivered financial and social incentives on cooperation [16,19].

Concerning rewards and punishments, evidence from the literature on centralized incentives is mixed, with some studies showing that centralized sanctions are more effective than centralized rewards [14,49], whereas others report the opposite pattern [47]. Here, we did not find differences between rewards and punishments, which is in line with findings indicating that rewards and punishments of equivalent magnitudes likely have similar effects on cooperation [2].

Although not hypothesized, we found that the size of the initial endowment impacted participants’ willingness to cooperate, namely that the lower the initial endowment participants received, the more they (proportionately) contributed to the group pot. Similar results have been reported in an earlier study from our group [50] and might be explained in light of previous work on risk aversion demonstrating that people become more conservative in choosing lotteries as possible payoffs increase [41]. In the present study, higher initial endowments also represented the possibility of higher payoffs, and so participants may have become more risk averse, that is, deciding to contribute less to the group pot and subsequently increasing the certainty of their payoffs by relying less on their counterparts’ decisions.

Importantly, despite the complexities of the task that participants performed, the replication of our results across two different samples of participants and across two different testing contexts (i.e., behavioral laboratory and fMRI scanner) minimizes the possibility that results are due to participant confusion or lack of understanding of the instructions. In addition, we believe that the observed differences between conditions would have been unlikely if participants had issues in understanding the task. Instead, our behavioral results are robust and speak in favor of the fact that individuals discriminate between different types of centralized incentives when deciding whether or not to cooperate.

In addition to the behavioral findings, the neuroimaging results provided important clues as to how financial and social incentives impact the psychological mechanisms underlying cooperation. We observed an increase in activation in lOFC when incentives were absent as compared to when they were present. Previous work indicates that the lOFC is associated with the processing of social norms; for example, patients with OFC lesions display inappropriate behavior, which is hypothesized to result from a failure to compare one’s behavior to internalized social norms [51]. More specifically, the lOFC is involved in detecting potential punishments and in turn generating signals that will ultimately lead to a necessary change in behavior to avoid these punishments [52]. In the present study, the decision to cooperate in the absence of incentives could be interpreted as having greater reliance on internalized social norms (e.g., the anticipation of feelings of guilt if you do not meet the expectations of others) than when incentives were present. In the absence of incentives, participants may have felt more uncertain about the behavior of their counterparts in the game and indeed about what was expected from them. This lack of clarity about one’s “optimal” decision likely creates a greater necessity to adhere to one’s internal social norms. In contrast, the provision of incentives may have decreased decision uncertainty by providing more explicit cues as to the “proper” game behavior, thus decreasing the necessity to rely on internal social norms.

Next, our neuroimaging results showed that at the moment of the decision itself, activation in the precuneus was greater in the absence as compared to the presence of incentives. Precuneus activation has been linked to the theory of mind network, as reflected in a previous meta-analysis and review [53,54]. Importantly, studies have reported greater precuneus activation when one engages in third-person perspective simulation in comparison to first-person perspective taking [55,56]. As the precuneus is involved in other cognitive processes it is important to be cautious while interpreting its activation here; nevertheless, one possible explanation for its stronger activation when incentives are absent is that in this scenario, participants are more likely to engage in self-other oriented thoughts, including mentalizing and strategizing about the behavior of others.

Indeed, our behavioral data support the role of perspective-taking processes on cooperative behavior by showing that participants’ contributions positively correlate with self-reported expectations about others’ choices in the PGG. Although self-reported expectations may be biased by one’s own contribution choices in the game, we believe this finding provides further evidence that people do attend to social norms of conditional cooperation [6], and therefore expectations about how others will behave do indeed matter in behaving cooperatively.

Neuroimaging results also showed stronger activation of the medial orbitofrontal cortex (mOFC) for social versus financial incentives. Activation in this region has previously been associated with both subjective value [57] and social cognition [30]; therefore, this finding could suggest that to some extent, social incentives are processed differently than financial incentives. Interestingly though, such differences did not arise at an earlier stage, when participants were informed about what type of incentive could potentially be received in a given trial. This suggests that the impact of social incentives is strongest during the moment of decision-making itself. In addition, it is noteworthy that the activation of the mOFC is not modulated by participants’ responses. Indeed, the behavioral effect seen in our study was the opposite from the neural pattern, as financial incentives yielded the highest contribution rates in the PGG. This seemingly inconsistent pattern between behavioral and imaging results might suggest that activation of the mOFC during the provision of social incentives reflects socio-cognitive processes that may be more strongly elicited during social incentives than financial incentives, which is possibly due to the greater salience of social approval and social comparison processes elicited in the presence of social incentives. Although caution is warranted when inferring psychological processes from brain activity, this interpretation is in line with previous studies showing activation in the medial prefrontal cortex when individuals are exposed to situations in which their decisions can be evaluated by their peers [58] and when social comparison is salient [59]. Further investigation of the neural mechanisms of financial and social incentives is important in order to advance our understanding about the similarities and differences between these two effective forms of incentives.

In general, we believe the present study provides some interesting avenues for future exploration. For example, future studies could directly compare the effect of centralized and peer-to-peer incentives on cooperation and its underlying neural mechanisms, following this and other studies [15,60,61]. In addition, higher activation of bilateral OFC has been previously reported when participants faced the threat of punishment, in comparison to facing no incentives [32]. This contrasting finding is likely due to differences in experimental design, such as the use of peer-to-peer incentives versus centralized punishment, as well as the inclusion of different types of incentives (both financial and social incentives as well as rewards and punishments) in the current study. Therefore, another potentially interesting future direction is to explore whether incentives affect psychological and neural processes involved in cooperation differently over time, for example, before and after individuals are informed about actually receiving the incentive. To sum up, our neuroimaging results suggest that the need to rely on internal social norms and perspective-taking processes could be reduced when incentives are present. The provision of centralized incentives might make one’s own and (others’) expected behavior clearer, reducing the need to access internal motivations in order to decide whether or not to cooperate. This is an intriguing inference we can make from the brain data, as while centralized incentives may well work to “nudge” behavior in desired directions, it may have this effect via effectively reducing the degree to which people utilize prosocial motivations, suggesting an interesting “boomerang” effect worthy of future study.

The present results not only enrich the literature on incentives and cooperation by providing a better understanding of the neural mechanisms underlying cooperative behavior and how these mechanisms can be affected by external centralized incentives, but they also help illuminate the design of policy campaigns aiming to increase cooperation [62,63]. For instance, when the use of centralized incentives is not possible, public campaigns could boost internal motivations to cooperate, such as wearing a facemask during a pandemic, by targeting perspective-taking processes and social norms of cooperation. In addition, our results also point out the relevance of social incentives. Although centralized social incentives are employed much less often than centralized financial incentives, our results suggest that the former could play a more prominent role in the central authority toolbox, potentially even serving as an alternative to financial incentives, given that they are somewhat comparable in efficacy but are undoubtedly less costly to implement [64].

## Figures and Tables

**Table 1:** Brain activations related to the anticipation model (GLM 1, time-locked to screen II). All reported activations survived a threshold of *p* < 0.05, family-wise error (FWE) corrected for multiple comparisons at the cluster level, with an underlying threshold corresponding to *p* < 0.001 uncorrected at the whole-brain level. Abbreviations: L/R: left/right; *x*, *y* and *z*: coordinates in Montreal Neurological Institute (MNI) space.

Brain Region	Hemisphere	*x*	*y*	*z*	Number of Voxels	Z Score (max)	FWE corr.
*No incentives* vs. *incentive*							
Lateral orbitofrontal cortex	R	24	52	−8	128	5.45	*p* = 0.002
Lateral orbitofrontal cortex	L	−40	56	−8	74	3.93	*p* = 0.022

**Table 2:** Brain activations related to the decision-making model (GLM 2, time locked to screen V). All reported activations survived a threshold corresponding to *p* < 0.05, FWE corrected for multiple comparisons at the cluster level, with an underlying threshold corresponding to *p* < 0.001 uncorrected at the whole-brain level. Abbreviations: L/R: left/right; *x*, *y*, and *z*: coordinates in MNI space.

Brain Region	Hemisphere	*x*	*y*	*z*	Number of Voxels	Z Score (max)	FWE corr.
*No incentives* vs. *incentive*							
Precuneus	L	−8	−52	48	223	4.69	*p* < 0.001
*Social* vs. *financial incentives*							
Medial orbitofrontal cortex	R	6	24	−22	110	4.4	*p* = 0.013

## Data Availability

The datasets generated during and analysed during the current study are available from the corresponding author on request.
